# A portable neurostimulator circuit with anodic bias enhances stimulation injection capacity

**DOI:** 10.1088/1741-2552/ac8fb6

**Published:** 2022-10-05

**Authors:** Alpaslan Ersöz, Insoo Kim, Martin Han

**Affiliations:** 1 Department of Biomedical Engineering, University of Connecticut, Storrs, CT, United States of America; 2 Department of Medicine Division of Occupational and Environmental Medicine, University of Connecticut, Farmington, CT, United States of America

**Keywords:** anodic bias, charge injection, embedded neurostimulator system, iridium oxide

## Abstract

*Objective.* Electrochemically safe and efficient charge injection for neural stimulation necessitates monitoring of polarization and enhanced charge injection capacity of the stimulating electrodes. In this work, we present improved microstimulation capability by developing a custom-designed multichannel portable neurostimulator with a fully programmable anodic bias circuitry and voltage transient monitoring feature. *Approach.* We developed a 16-channel multichannel neurostimulator system, compared charge injection capacities as a function of anodic bias potentials, and demonstrated convenient control of the system by a custom-designed user interface allowing bidirectional wireless data transmission of stimulation parameters and recorded voltage transients. Charge injections were conducted in phosphate-buffered saline with silicon-based iridium oxide microelectrodes. *Main results.* Under charge-balanced 200 *µ*s cathodic first pulsing, the charge injection capacities increased proportionally to the level of anodic bias applied, reaching a maximum of ten-fold increase in current intensity from 10 *µ*A (100 *µ*C cm^−2^) to 100 *µ*A (1000 *µ*C cm^−2^) with a 600 mV anodic bias. Our custom-designed and completely portable 16-channel neurostimulator enabled a significant increase in charge injection capacity *in vitro. Significance.* Limited charge injection capacity has been a bottleneck in neural stimulation applications, and our system may enable efficacious behavioral animal study involving chronic microstimulation while ensuring electrochemical safety.

## 1. Introduction

Neural activation using microelectrodes such as in intraspinal stimulation [1], cochlear nucleus auditory prosthesis [2–4], and visual prosthesis [5, 6] requires high charge injection capacity to obtain desired functional and behavioral effects. Although the microelectrodes, generally with geometric surface areas (GSAs) of 500–10 000 *µ*m^2^, are designed for high selectivity, spatial resolution, and scalability in depolarizing large ensembles of neurons, they may surpass safe electrochemical ranges before achieving neural activation thresholds *in vivo* [7]. Thus, expanding the therapeutic window of neural activation while avoiding electrode and tissue damage is paramount.

Previous efforts in enhancing charge injection capacity primarily focused on new electrode materials [8–11] and enlarging effective surface areas [12, 13]. However, improvements *in vivo* have been lagging. For example, platinum (Pt) electrodes had approximately nine times less charge injection capacity *in vivo* than *in vitro* (with 200 *µ*s pulse widths) [13]. Diamond [14] and graphene [15] also showed similar or lower charge injection capacity than Pt [15].

An alternative technique to increase the charge injection capacity of the microelectrode is by applying an anodic bias potential [16–20]. It is known that certain microelectrodes become more conductive when their initial potential is elevated (mostly 0.4 V–0.8 V vs. Ag|AgCl), and iridium oxide electrodes (IrO*_x_*) in particular become more conductive by conditioning Ir^3+^ into Ir^4+^ valence state [8]. Advantages of anodic bias potential include: (a) it may work on multiple types of electrode materials, (b) it does not require modifications on electrodes, (c) its bias levels can be readily adjusted, (d) it facilitates control of voltage transients in safe ranges, and (e) it enables a greater swing in cathodic pulse intensity which is known to excite neural tissues. The charge injection enhancement with anodic bias has been validated using various materials in both *in vitro* and *in vivo* experiments. Cogan *et al* [16], in an *in vitro* study with activated iridium oxide film (AIROF) microelectrodes, found that a 0.6 V anodic bias voltage enhanced the mean charge injection capacity of the electrode by 63%. Deku *et al* reported enhancement of charge injection capacity of TiN using anodic bias [21]. Han *et al* [22] and McCreery *et al* [23], and Troyk *et al* [24] demonstrated *in vivo* effective charge injection through electroplated IrO*_x_* and AIROF microelectrodes for stimulation of the cat brain and rat sciatic nerve, respectively.

Previously, bias voltage was often set by operating an analog potentiometer with feedback circuitry in the pulse generator to control an offset voltage across the stimulating electrodes [17, 18]. However, analog potentiometers with carbon-composition resistors (having an error tolerance of at least 1% [25]) and a rotatable contact with manual control have low voltage adjustment precision. Therefore, digitally programmable anodic bias control will provide better accuracy and finer dosages which is important in microstimulation. Moreover, a wireless bidirectional mode (both transmission of stimulation waveforms and reception of voltage transients) will be desired in awake behaving animal experiments.

We have designed a novel, digitally controlled neurostimulator system with commercially available off-the-shelf components [26]. In this work, we present an upgraded and fully-portable embedded neurostimulation system that wirelessly delivers constant current stimulation signals with or without anodic bias to 16 channels and records voltage transients to monitor electrochemical safety. Validation of the system was accomplished with a custom-designed IrO*_x_* microelectrode probe [27] through extensive *in vitro* experiments in which anodic bias was shown to significantly enhance charge injection capacity (*Q*_inj_).

## 2. Materials and methods

### 2.1. Hardware

**Figure 1.**
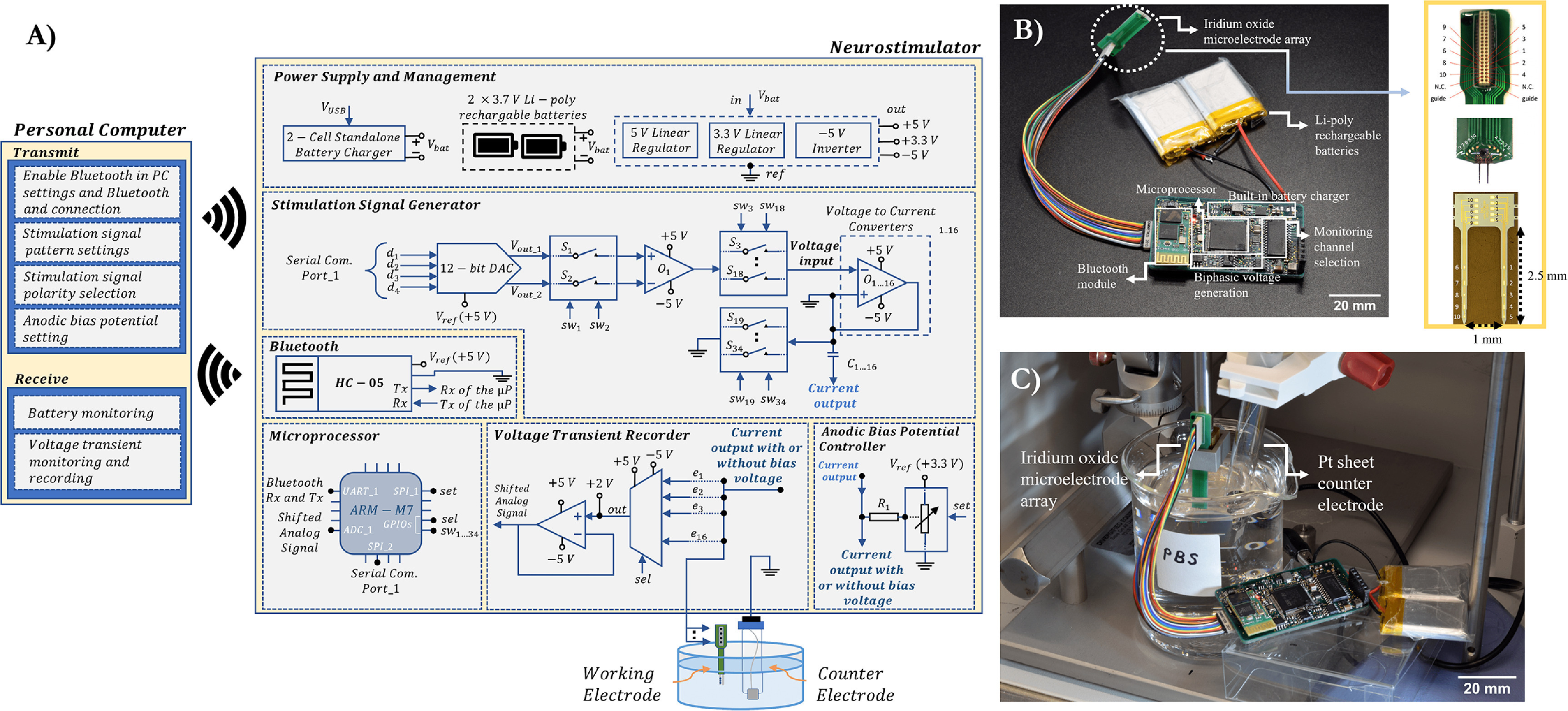
The neurostimulator and *in vitro* test setup. (A) System-level block diagram of the neurostimulator electronics circuit and the user interface. (B) Assembled and electrically tested neurostimulator with rechargeable batteries and microelectrode PCB consisting of microelectrode array and Omnetics connector. (C) *In vitro* setup of the neurostimulator with the microelectrode PCB in a Faraday cage. Abbreviations: Li-Poly: lithium polymer, DAC: digital-to-analog converter, SW: switch, UART: universal asynchronous receiver-transmitter, SPI: serial peripheral interface, GPIO: general purpose input/output, Rx: reception of data, Tx: transmission of data, *µ*P: microprocessor, Pt: platinum, N.C: not connected, PBS: phosphate-buffered saline.

The schematic diagram of the neurostimulator is shown in figure 1, and its main specifications are listed in table 1. The system consists of a user interface in the personal computer and the neurostimulator circuit consisting of six subblocks: microprocessor, power supply and management, stimulation signal generator, anodic bias potential controller, voltage transient recorder, and a Bluetooth module.

**Table 1.** Main specifications of the neurostimulator system.

Features	Details
Batteries	Two Li-Poly 3.7 V 500 mA cells (rechargeable)
Supply voltage	7.4 V
Programmable current level	1–160 *µ*A
Stimulation channels	16
Voltage monitoring channels	16
Pulse pattern	Monophasic/biphasic symmetric/asymmetric
Anodic bias control	Programmable, 0–3.3 V
Pulse width	1–2000 *µ*s (resolution 1 *µ*s)
Pulse polarity	Cathodic or anodic first
Data transmission	Wirelessly with Bluetooth SPP module
Voltage compliance	±5 V
Dimensions	35.82 mm (width) × 59.06 mm (length)

#### 2.1.1. User interface

**Figure 2.**
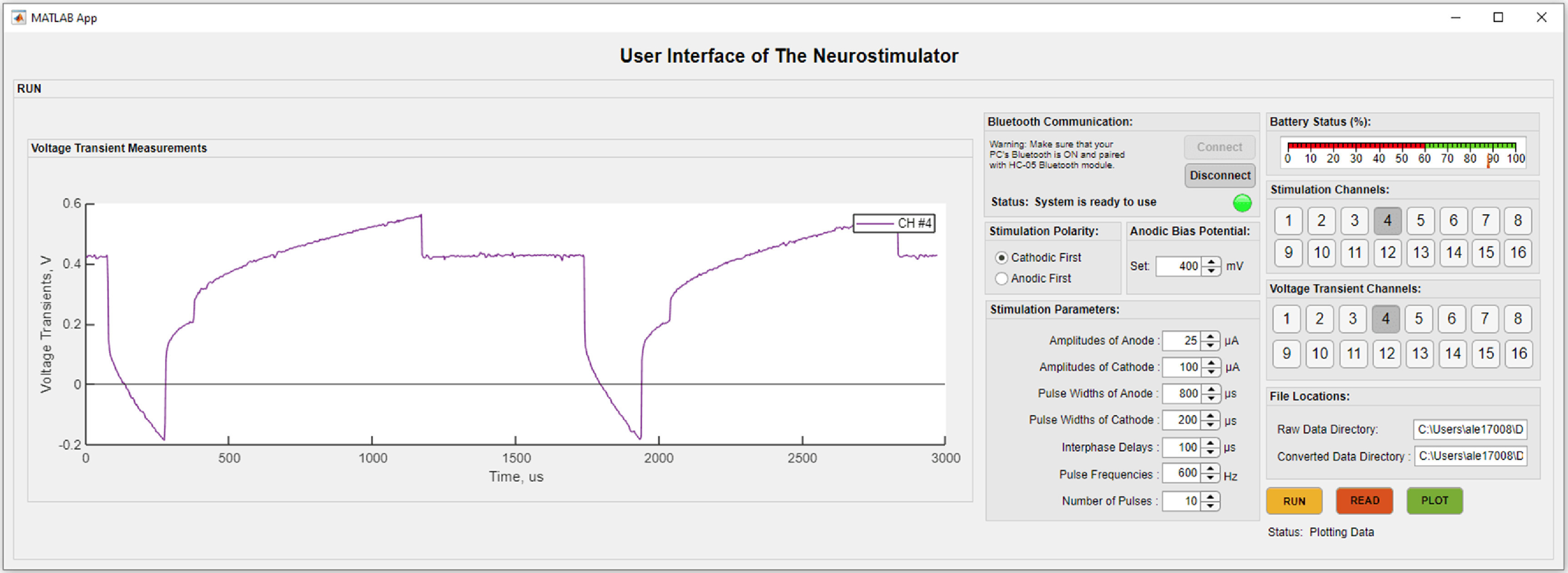
A user interface was developed in MATLAB app designer. The interface allows setting stimulation waveforms parameters such as amplitude, pulse widths, interphase delay, frequency, signal polarity, and anodic bias. It also plots voltage transients and battery status, and handles data files.

An intuitive user interface system (figure 2) was developed in MATLAB App Designer which can be installed as a stand-alone executable program. The interface allows setting stimulation waveforms such as amplitude, pulse width, interphase delay, frequency, signal polarity, and anodic bias*. Bluetooth Communication* establishes a connection with the Bluetooth module. Stimulation polarity specifies pulse phases. *Anodic Bias Potential* conveniently sets bias levels. *Stimulation Channels* selects either single channels or multiples at once; *Voltage Transient Channels* select which channels to acquire voltage transient data. *Run* initiates transmission of command signals to the processor and storing of voltage transients and battery information in a raw 12-bit format in the PC. Read converts the raw data to true voltage values which then can be exported to different data processing software. *Plot,* for convenience, displays voltage transients in the interface window as shown in figure 2 (left), and updates *Battery Status.*

#### 2.1.2. Neurostimulator circuit

A 32-bit ARM cortex M-7 microprocessor (STM32F767 VI) processed binary data acquisition, saved the digitized data into its internal flash memory, and implemented system logic. The microprocessor and other chips (or integrated circuits (ICs)) were powered by rechargeable batteries. A pack of two lithium-polymer batteries in series (Akzytue 602 535), provided 500 mAh current capacity and 7.4 V (8.4 V when fully charged). To regulate the battery output voltage into required voltage of system’s circuitry, linear regulators were incorporated. The low drop-out voltage regulators (LT1763-3.3 and LT1763-5) provide constant 3.3 V and 5 V outputs with low noise. A stepdown voltage converter (LM43601) inverts the battery outputs to −5 V for feeding the rail-to-rail power inputs of the operational amplifiers (op-amps), multiplexer, and switches. At the power inputs of each IC on the circuit, 0.01 *µ*F decoupling capacitors are kept close (within 0.5 inches of the IC [28]) to the ICs to smoothen high-frequency changes in the power supply and provide immediate electrical energy demands to maintain stable voltage supply. When the battery level drops below 2.75 V, the batteries are charged by an external 5 V DC power supply through a USB 2.0/3.0 cable using the on-circuit highly-integrated switch-mode battery charge management IC (BQ25886RGER) and peripherals. Charging ports (GND and 5 V) were designated within the Omnetics pins.

The microprocessor enables an external 12-bit rail-to-rail digital-to-analog converter (DAC) (DAC122S085) with an serial peripheral interface (SPI) interface. The DAC can vary analog voltage output up to 5 V and generates two separate step-function signals based on processor commands. The microprocessor controls the two single-pole single- throw (SPST) switches (S1 and S2) through assigned general purpose input/output pins that convert the step function signals to monophasic signals. The monophasic signals represent the anodic and cathodic phases of a biphasic waveform. Op-amp-based voltage subtractor circuit operates with a gain of two to get a biphasic voltage signal from the monophasic pulses. Temporal parameters such as pulse width, interphase delay, and frequency are set by the duration of ON and OFF periods of the S1 and S2 switch operations.

The biphasic voltage signals are fed into the input of the Howland current circuits via 16 separate SPST switches (S3–S18) simultaneously. The remaining SPST switches (S19–S34) are responsible for shorting the Howland current pump outputs coupling capacitor (C1…16) to the system ground after the end of each stimulation pulse. Connecting a coupling capacitor before charge injection to the electrodes blocks any DC flow under system fault, corrects charge imbalance, and limits excessive net charge delivery to the tissues [29].

Anodic bias potential is applied through a high-impedance current-limiting resistor (*R*1) connected directly to the electrode. The bias potential is set through an SPI interfaced digital potentiometer (MCP41100, Microchip Technology, USA). The potentiometer voltage input has a constant of 3.3 V, and the output bias voltage is generated depending on the wiper position of the potentiometer which is updated by 8-bit (256 positions) potentiometer’s data register from the microprocessor. The digital potentiometer has 13.3 mV precision and can generate output voltage between 0 V and 3.3 V.

Voltage transients during constant-current stimulation are used to estimate the level of polarization on the electrode beyond which irreversible electrochemical damage may occur. Transients are obtained with a 16 × 1 analog multiplexer and a non-inverting level shifter which adjusts the raw voltage values to 0–3.3 V range. An internal successive-approximation register (SAR) analog-to-digital converter (ADC) (12-bit, 2.4-MSpS) digitizes the voltage transients with a 354 kSpS sampling rate and saves them into the processor’s flash memory.

A Bluetooth module is interfaced with the microprocessor through a serial communication port in asynchronous mode. The baud rate of the module is set at 9600, and is capable of up to 3 Mb s^−1^ data transmission rate. Due to the serial communication structure of the Bluetooth, while 16 stimulation channels can enable simultaneously, the voltage transients are received sequentially using a 16 × 1 multiplexer.

### 2.2. Firmware

The microprocessor is programmed to execute stimulation and digitize recorded signals. A 96 MHz core clock signal is configured using an external clock, internal phase-locked loops, and frequency dividers, generating a stimulation signal with a resolution of 1 *µ*s. The programming is written in C language using Keil *µ*Vision 5.26 IDE, and the processor’s pins are designated with STM32CubeMX toolchains. The embedded controller allows 1–160 *µ*A current injection and chooses pulse widths of 1–2000 *µ*s with a 1 *µ*s resolution. Programming of the controller is established through an ST-Link adapter. The front end of the adapter is connected to a serial wire debugging connector on the printed circuit board (PCB), and the back end of the adapter is linked to a personal computer via a USB port.

### 2.3. Cable and connector system

The neurostimulator system and the microelectrode probe were connected to each other with a ribbon cable that had two female Omnetics connectors at its front and back end. Each row of the female Omnetics connector had an 18-conductor cable with a low resistance conductivity. The neurostimulator system has a horizontal male Omnetics connector, and the microelectrode probe PCB has a vertical male Omnetics connector. The connector type on the neurostimulator system, microelectrode probe PCB, and jumping cable is nano miniature Omnetics and has 36 pins. About 16 of the Omnetics connector pins on the neurostimulator were designated for stimulation and voltage transient monitoring, four for returning paths (GND), and two for USB 5 V and GND of the on-circuit battery charger system, and the rest were unused.

### 2.4. Validation

Bench-top electrical tests and *in vitro* experiments were used to test the functions of the neurostimulator system which was fabricated and assembled on a 6-layer PCB. In the initial electrical tests, 10 kΩ, 20 kΩ, and 30 kΩ resistors were connected to stimulation channels as a working electrode output to ascertain the current conversion from biphasic voltage transient waveforms. The working electrode was a custom-designed activated IrO*_x_* microelectrode probe with 2000 *µ*m^2^ GSA [27].

An electrochemical impedance spectroscopy measurement was taken in phosphate buffered saline solution using Autolab PGSTAT128N (Metrohm AG, Switzerland) with 10 mV-rms sinusoids. A three-electrode configuration was used. The working electrode was our microelectrode probe, the counter electrode was a platinum sheet electrode (Metrohm), and the reference electrode was Ag|AgCl electrode (FisherScientific, NH).

In the charge injection experiments, a two-electrode configuration was used. The working electrode was the microelectrodes connected to the stimulation channels, and a Pt sheet counter electrode connected to the system ground (figure 1(C)). Stimulation current signals were charge-balanced biphasic asymmetric signals with a cathodic-first polarity. The pulse width ratio of the stimulus patterns was set at 1:4 cathodic- to-anodic, and their amplitudes were at 4:1. The biphasic signals were set from 1 *µ*A to 160 *µ*A, and the bias potential was programmed from 100 mV to 600 mV, to determine improvements in charge injection capacities. The interphase pulse widths of the biphasic signals were 100 *µ*s, and their pulse repetition frequencies were set at 625 Hz.

## 3. Results

The neurostimulator circuit was composed of 344 surface mountable device components which were soldered onto a 59.06 × 35.82 mm custom PCB (figure 1(B)). Overall weight, including batteries, was 35.4 g. Electrical conductivity, mechanical strength, and soldering quality tests of the assembled components were completed before flashing the system. Once stimulation parameters were received by the neurostimulator circuit, the microprocessor went into the working mode, and transmitted converted data to the PC (figure 2). Based on the plotted voltage transients in the interface, the anodic bias voltage can be adjusted to increase charge injection capacity. Voltage transient and battery monitoring results were saved in a directory in the PC, and were plotted in the interface as shown in figure 2.

**Figure 3.**
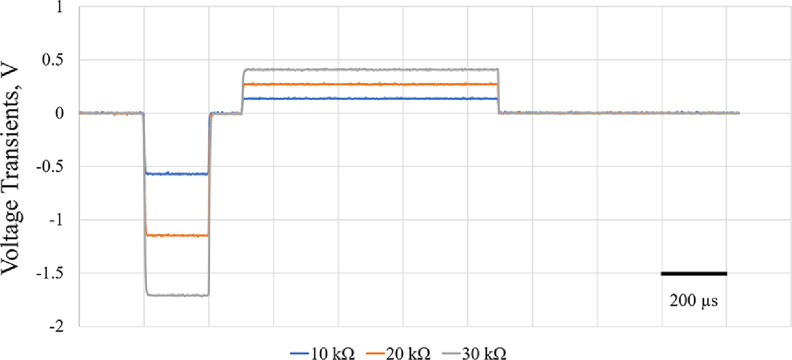
Voltage transients of 60 *µ*A cathodic first biphasic asymmetric (1:4 cathodic-to-anodic pulse width ratio and 4:1 cathodic-to-anodic amplitude ratio) constant current stimulation. Three different intensity loads (10 kΩ–30 kΩ) were connected as working electrode for validation of the current intensity output of the system.

Initial electrical tests of the neurostimulator system using simple resistors were conducted to observe signal generation while monitoring voltage transient response on different loads. Biphasic 60 *µ*A constant current stimulation signals were injected, and voltage transients in response to the stimulation signal were monitored wirelessly (figure 3). The voltage transient peaks for cathodic phases were recorded as −0.57 V, −1.15 V, and −1.71 V, respectively.

**Figure 4.**
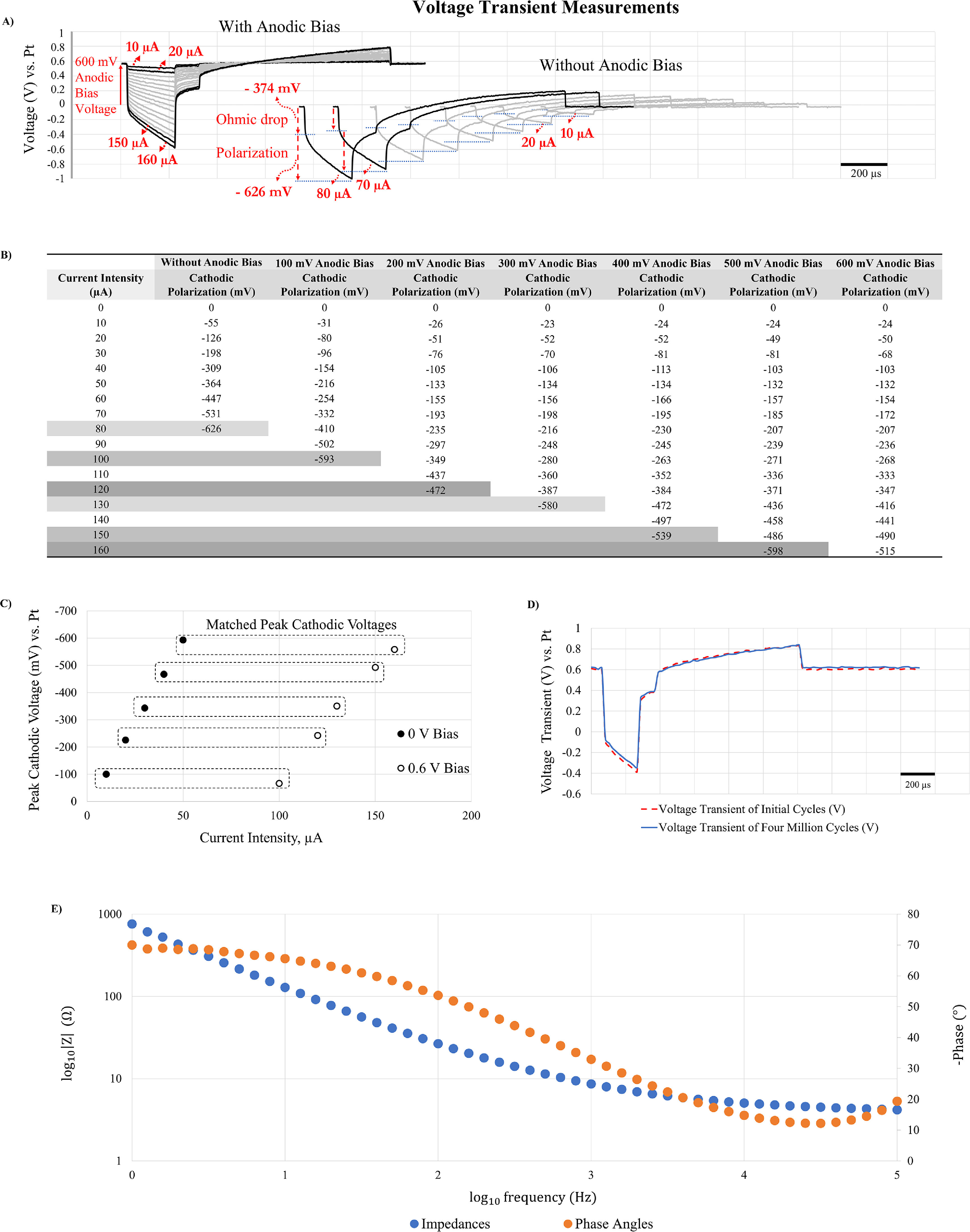
Demonstration of charge injection enhancement using anodic bias applied through the neurostimulator circuit via telemetry. (A) Comparison of voltage transients in response to constant current stimulations with a 600 mV bias potential (Left) and without anodic bias potential (Right). Anodic bias helped inject twice the current intensity (160 *µ*A vs. 80 *µ*A). (B) Cathodic polarization voltages in response to increasing current intensities and under varying anodic bias potentials. Anodic bias enabled greater current injection. For instance, without anodic bias 80 *µ*A was the maximum intensity, whereas with 500 mV or 600 mV bias, the current injection limit was safely extended to 160 *µ*A. (C) Pairing of current intensities by peak cathodic voltages shows three to ten fold increases in charge injection with the use of 600 mV anodic bias. (D) Comparison of initial and after 4 million cycles of continuous stimulation with 600 mV anodic bias voltage with 120 *µ*A. After 4 million cycles, the voltage transient of the IrO*_x_* electrode and anodic bias potential were stable which shows robustness of the neurostimulator. (E) An impedance spectroscopy of an activated IrO*_x_* microelectrode, illustrating low impedance values and good electrochemical property.

*In vitro* voltage transients in response to the biphasic asymmetric constant current stimulation signals with and without anodic biases are shown in figure 4(A). It is noted that the voltage transient consists of the initial sharp voltage drop called ohmic drop and polarization. The former is also known as the access voltage which results due to resistance at the electrode-electrolyte interface, and does not cause electrochemical damages [30]. First, increasing current intensity resulted in greater ohmic drops and polarization, thus greater peak cathodic voltages. This happened regardless of whether anodic bias was applied (figure 4(A))-Left) or not (figure 4(A))-Right), which was as expected. A key difference was that without anodic bias potential applied, the current was limited at 80 *µ*A, since cathodic voltage transients extended to −1000 mV, of which −626 mV is due to the interface polarization. However, with a 600 mV anodic bias potential, the current intensity was doubled to 160 *µ*A without being restricted by expanding polarization voltages. Therefore, figure 4(B)) illustrates that applying a 600 mV anodic bias enhanced charge injection greatly, even leaving additional capability to inject more currents. Our stimulator’s maximum current output of 160 *µ*A is likely enough for most *in vivo* microstimulation applications in terms of activating neural tissues [7, 23, 31].

We studied the effect of varying levels of anodic bias on electrode polarization. Figure 4(B) shows that higher anodic bias resulted in less polarization, enabling more current to be injected. For example, a no-bias case reached its largest current at the earliest at 80 *µ*A, 100 mV of anodic bias at 100 *µ*A, 200 mV of anodic bias at 120 *µ*A, 300 mV of anodic bias at 130 *µ*A, 400 mV of anodic bias at 150 *µ*A, 500 mV of anodic bias at 160 *µ*A, and 600 mV of anodic bias also at 160 *µ*A. Therefore, there was an inverse relationship between the bias level and polarization, and a direct relationship between the bias level and current intensity. In other words, the use of anodic bias allowed maximization of current injection while reducing polarization, thus safer electrochemical dynamics.

Stimulation parameters that resulted in approximately the same values of peak cathodic voltages are illustrated in figure 4(C). A no-bias 10 *µ*A and 600 mV-biased 100 *µ*A had similar peak cathodic voltages. Thus, in this case, the current intensity was increased by a factor of approximately ten (from 10 *µ*A to 100 *µ*A). Similarly, no-bias 20 *µ*A and 600 mV-biased 120 *µ*A had similar peak cathodic voltages, thus a six-fold increase in the current intensity; no-bias 30 *µ*A and 600 mV-biased 130 *µ*A had an approximately four-fold increase; both no-bias 40 *µ*A and 600 mV-biased 150 *µ*A and no-bias 50 *µ*A and 600 mV-biased 160 *µ*A had approximately three-fold increases. Thus, the charge injection capacity of the AIROF microelectrodes was enhanced by a factor of ten at the maximum and a factor of three at the minimum, depending on a range of the current intensity. In all cases, the voltage transients did not exceed the limits of the cathodic electrolysis.

The consistency of the neurostimulator and property of the IrO*_x_* microelectrode array were evaluated with 4 million continuous cycles of pulses (figure 4(D)). The electrode was subjected to continuous biphasic current pulsing at 600 mV anodic bias and 24 nC/phase (120 *µ*A current intensity, 200 *µ*s cathodic phase pulse width; 100 *µ*s interphase pulse width; 800 *µ*s anodic phase pulse width; 500 *µ*s off-period), at a frequency of 500 pulses per second for a total of approximately 33 min. Voltage transients before and after 4 million cycles were very similar which demonstrated the reliability of the neurostimulator in continuous stimulation mode. Figure 4(E) shows an AC impedance plot of activated IrO*_x_* microelectrode. At 1 kHz, the impedance value was 8.64 kΩ at 1 kHz which is very low, illustrating a suitable electrochemical property.

## 4. Discussions

Our embedded neurostimulator system, designed with discrete components, demonstrated a battery-powered, low-cost, and wirelessly controlled multichannel electrical stimulation with programmable charge injection enhancement *in vitro*.

Table 2 provides a comparison between our system and other neurostimulators. Previous studies used a non-programmable charge injection (manually with an analog potentiometer) on a single channel and external oscilloscope to monitor voltage responses [17, 18]. Our system offers unique and key combinations of features such as programmable anodic bias control, multichannel constant-current stimulations, and voltage transients monitoring with a built-in wireless bidirectional data transmission. These advantages may prove important for freely behaving animal studies.

**Table 2.** Comparison to other systems in the literature.

Features	Kölbl *et al* [40]	Fluri *et al* [41]	Kouzani *et al* [42]	Adams *et al* [43]	Elyahoodayan *et al* [44]	This Work
Anodic bias potential	No	No	No	No	No	Digital, 0–3.3 V
Stimulation	Bilateral, constant-current	Single channel, constant-current	Single channel, constant-current	Single channel, constant-current	32 Channel, constant-current	16 Channel, simultaneous constant-current
Built-in voltage transient monitoring	N/A	No	N/A	No	No	Yes
Sampling rate	N/A	N/A	N/A	External device with 50 MS s^−1^	External device with 100 kS s^−1^	354 kS s^−1^
Pulse width	60 *µ*s	60–500 *µ*s	90 *µ*s	20–155.7 *µ*s	1–200 *µ*s	1–2000 *µ*s
Wireless data transmission	No	No	Yes	No	No	Yes
User interface	No	No	No	No	No	Yes
Current intensity	15–1600 *µ*A	10–500 *µ*A	200 *µ*A	0–200 *µ*A	1–60 *µ*A	1–160 *µ*A
Battery	Powerone Zinc/air p675 1.45 v/650 mAh	Single coin cell 1.55 V/20 mA	Single coin cell 3.7 V/250 mA	Single coin cell 3.7 V/220 mA	Two-coin cells 3.7 V/[N/A] mA	Two Li-poly batteries 3.7 V/550 mAh and on-circuit charging

We can currently use the electronics in benchtop or intraoperative settings, but a longer-term purpose of implementing the Bluetooth interface would be for use in freely-behaving small animals (although this utility is not a main focus of this report). The current physical dimension may be small and light enough for the rats as a backpack-worn stimulator as similarly done in a recent long-term stimulation safety study in the cats [23]. There are several benefits of our wireless system, including: (a) stimulation parameterization and data transfer without restricting animal mobility extracorporeally and risking connector failure after repeated mating, (b) reduced tethering of animal’s body to the instrument which may introduce less stress during long-term stimulation experiments and, thus, enhance signal fidelity and reproducibility, and (c) improved scalability of stimulation channels without adding bulkiness to the cables and connectors.

Having many features requires extensive hardware and firmware designs. Existing neural interface systems [32–34] have generally been designed as either separated modules (neurostimulator and hub system) or interconnected printed circuit boards. Separated modules require individual power units, microcontrollers, and connectors to link the modules. Therefore, a compact design is desired for freely moving animal experiments, avoiding possible connection failures among PCB modules, complex firmware programming, and short battery life. We developed a single printed circuit board-based neurostimulator system that simplified such issues while allowing for efficient stackup layer plan and crosstalk and coupling elimination. Application-specific IC-based neurostimulators can further miniaturize overall form-factors [35–37].

We noticed that the access resistance, calculated from the ohmic voltage drop (*R* = *V*/*I*) of figure 4(C), decreased as the anodic bias increased, from 3.80 kΩ (*R* = 38 mV/10 *µ*A) at 0 V to 3.36 kΩ (*R* = 336 mV/100 *µ*A) at 600 mV of anodic bias. This supports our hypothesis that the anodic bias makes the IrO*_x_*’ valence states more conductive, thus resulting in lower interfacial resistance.

*In vivo* charge injection enhancement with anodic bias potential was validated by our group and others previously [20, 22, 23]. They demonstrated that electronics-based charge enhancement was efficient and convenient in *in vivo* animal studies. Regarding safety, during the off-duty period, a negligible amount of external current flowed through the microelectrode (<0.01 *µ*A). This ensures that the system avoids injecting extra net charge into the electrolyte or tissues, potentially resulting in tissue damage *in vivo*. Our recent work involving a 400 mV-anodic bias in a long-term *in vivo* study reported that intracortical stimulation of feline pyramidal neurons for 20 d resulted in minimal damage compared to control tissue [23].

In the testing under this study, we applied the anodic bias against Pt electrode. We measured an approximately 270 mV of open circuit potential for Pt vs. Ag|AgCl using AutoLab instrument, thus we believe that among the anodic bias potentials tested in this study (i.e. 100 mV, 200 mV, 300 mV, 400 mV, 500 mV, and 600 mV), 600 mV is likely be an upper limit as the actual electrode potential (600 mV + 270 mV = 870 mV) is at the onset of water oxidation. While we may be underutilizing charges in the cathodic region, IrO*_x_* is known for potential damages beyond −0.6 V (vs. Ag|AgCl; Cogan 2008), and we are already injecting a maximum cathodic-first current intensity afforded by our stimulator which is likely be adequate for neural activation as noted.

In our preliminary study [38], we reported an increase of charge injection capacities of the Blackrock IrO*_x_* microelectrodes with 0.7 V anodic bias by nine-fold. In the report, we enhanced our custom multisite IrO*_x_* microelectrodes’ charge injection capacity with 0.6 V anodic bias by a maximum of ten-fold. In both cases, we calculated the increases in current injection using cathodic polarization voltages. In this study, the enhancement, however, was reduced from ten-fold to approximately three-fold in larger current intensity pairs (figure 4(C)). The data collectively, however, illustrate that the improvement is independent of device styles (single tip Blackrock electrodes vs. multisite silicon array). In addition, charge injection enhancement is not limited only to the IrO*_x_* microelectrodes that were used in this study. Other materials such as Pt/Ir [8], Ta/Ta_2_O_5_ [39], and PEDOT [8] also respond to anodic bias, providing improvement in charge injection capability. Repetitive anodic biased charge injection, for instance, using 4 million cycles as done in this study or more, may confirm their long-term suitability for chronic microstimulation.

## 5. Conclusion

This study showed that charge enhancement of the IrO_x_ microelectrodes can be increased by a maximum of ten-fold with a digitally and wirelessly controlled anodic bias potential using a custom-designed multichannel neurostimulator system. In addition, to assure stimulation signal charge injection safety, the system can monitor and record voltage transient responses of the stimulation signals using a custom software interface. Enhancement of charge injection with anodic bias voltages has been demonstrated in vivo, in intracortical [23] and brainstem studies [22], paving the way for in vivo validation of our neurostimulator system.

## Data Availability

All data that support the findings of this study are included within the article (and any supplementary files).
